# Molecular hydrogen is a potential protective agent in the management of acute lung injury

**DOI:** 10.1186/s10020-022-00455-y

**Published:** 2022-03-03

**Authors:** Yan Zhang, Jin Zhang, Zhiling Fu

**Affiliations:** Department of Anesthesiology, Shengjing Hospital of China Medical University, Shenyang, 110004 People’s Republic of China

**Keywords:** Acute lung injury, COVID-19, Molecular hydrogen, ROS, Inflammation, Autophagy, Apoptosis, Pyroptosis

## Abstract

Acute lung injury (ALI) and acute respiratory distress syndrome, which is a more severe form of ALI, are life-threatening clinical syndromes observed in critically ill patients. Treatment methods to alleviate the pathogenesis of ALI have improved to a great extent at present. Although the efficacy of these therapies is limited, their relevance has increased remarkably with the ongoing pandemic caused by the novel coronavirus disease 2019 (COVID-19), which causes severe respiratory distress syndrome. Several studies have demonstrated the preventive and therapeutic effects of molecular hydrogen in the various diseases. The biological effects of molecular hydrogen mainly involve anti-inflammation, antioxidation, and autophagy and cell death modulation. This review focuses on the potential therapeutic effects of molecular hydrogen on ALI and its underlying mechanisms and aims to provide a theoretical basis for the clinical treatment of ALI and COVID-19.

## Background

The lungs not only are the primary location where gas exchange occurs in mammals but also have the largest epithelial surface in that is direct contact with the external environment. Therefore, the lungs are the primary target for many airborne pathogens, toxicants, and allergens that cause pneumonia, acute lung injury (ALI), and acute respiratory distress syndrome (ARDS) (Kumar 2020). ALI and its more severe form, ARDS, are life-threatening clinical syndromes that occur in critically ill patients (Hughes and Beasley 2017); these syndromes manifest as increased microvascular permeability, alveolar and interstitial edema with normal cardiac filling pressures, hyaline membrane formation, and atelectasis. Patients with ALI and ARDS exhibit symptoms such as acute hypoxic respiratory insufficiency or respiratory failure (Slutsky 2001). Despite advances in the treatment of preclinical and clinical conditions, the morbidity and mortality associated with ALI and ARDS are alarmingly high (Hayes et al. 2012), particularly since the outbreak of coronavirus disease 2019 (COVID-19) caused by severe acute respiratory syndrome coronavirus 2 (SARS-CoV-2) on December 31, 2019, in Wuhan, China, which subsequently resulted in a pandemic. Therefore, finding and developing new drugs so as to effectively treat ALI/ARDS is imperative; these may also be useful in the treatment of COVID-19.

In recent times, treatment to alleviate the pathogenesis of ALI has improved to a great extent, and existing therapies can be divided into supportive intervention and pharmacological treatment. Supportive intervention, such as lung-protective mechanical ventilation, together with adjunctive approaches, such as prone positioning and the use of neuromuscular blocking agents, has decreased mortality (Slutsky and Ranieri 2013). Pharmacological treatments involve the use of corticosteroids, antioxidants, neuromuscular blocking agents, fluid management, and stem cell-based therapeutics for treating influenza- and coronavirus-induced ALI (Shafeeq and Lat 2012; Du et al. 2020; Mrityunjaya et al. 2020); however, most have been unsuccessful till date (Villar et al. 2019).

Molecular hydrogen is colorless and odorless and the lightest chemical element in the earth’s atmosphere. For a long time, it was accepted that molecular hydrogen remains inert in mammalian cells. In 2007, Ohsawa et al*.* discovered that molecular hydrogen could selectively reduce hydroxyl radicals (**·**OH) and peroxynitrite (ONOO^−^), which are highly strong oxidants, in cells, thereby suppressing brain injury in ischemia/reperfusion (I/R) injury and stroke in a rat model (Ohsawa et al. 2007). In 2001, studies demonstrated that high-pressure molecular hydrogen gas has anti-inflammatory properties that can cure parasite-induced animal liver inflammation (Gharib et al. 2001). Subsequent cellular and animal studies as well as clinical experiments in diverse biomedical fields have demonstrated the preventive and therapeutic effects of molecular hydrogen in various organs, including the brain, heart, lung, pancreas, and liver, through its antioxidative stress, anti-inflammatory, antiapoptotic, and various other biological effects (Qiu et al. 2011; Hou et al. 2012; Xie et al. 2012; Luo et al. 2015; Liu et al. 2020b). The action of molecular hydrogen in the body is moderate and no side effects have been identified so far. It can be conveniently administered through various modes, including gas inhalation, hydrogen-rich saline (HRS) injection, hydrogen-rich water (HW), and as nanocrystals to achieve targeted delivery and controlled release (Kawamura et al. 2020). A simple method of administering molecular hydrogen is inhalation using a ventilator circuit, face mask, or nasal cannula. Hydrogen-dissolved water or HW is portable, safe, and easily administered. Molecular hydrogen can easily penetrate the skin, and a warm HW bath can be used therapeutically in daily life (Koyama et al. 2014; Ohta 2014). HRS injections may deliver more accurate molecular hydrogen doses (Ge et al. 2017). Molecular hydrogen utilized as nanoparticles exhibits high bio-reductivity and effective scavenging of cytotoxic OH (Zhang et al. 2019).

So far, the preventive and therapeutic effects of molecular hydrogen for various lung diseases, such as ALI, chronic obstructive pulmonary disease, asthma, and pulmonary arterial hypertension, have been extensively investigated (Kishimoto et al. 2015; Audi et al. 2017; Wang et al. 2020). China’s National Health Commission (7th trial ed NHC: Bejing, 2020) and the Chinese Center for Disease Control and Prevention (6th ed. CDCP: Bejing, 2020) have recommended effective oxygen therapy as a component of the general treatment for patients with COVID-19; they have also stated that a mixture of molecular hydrogen and oxygen (66.6% molecular hydrogen to 33.3% O_2_) for inhalation yields better results than oxygen alone (Conti et al. 2020; Ostojic 2020).

In this article, we summarize the most recently published literature concerning the application of molecular hydrogen for ALI caused by various pathological processes and briefly discuss the potential mechanisms underlying the action of molecular hydrogen in ALI. We hope that this review will facilitate our understanding of the therapeutic and preventive activities of molecular hydrogen and provide information that can be used to further the treatment of ALI and COVID-19 in the future.

## Pathophysiology of acute lung injury (ALI)

**Fig. 1:**
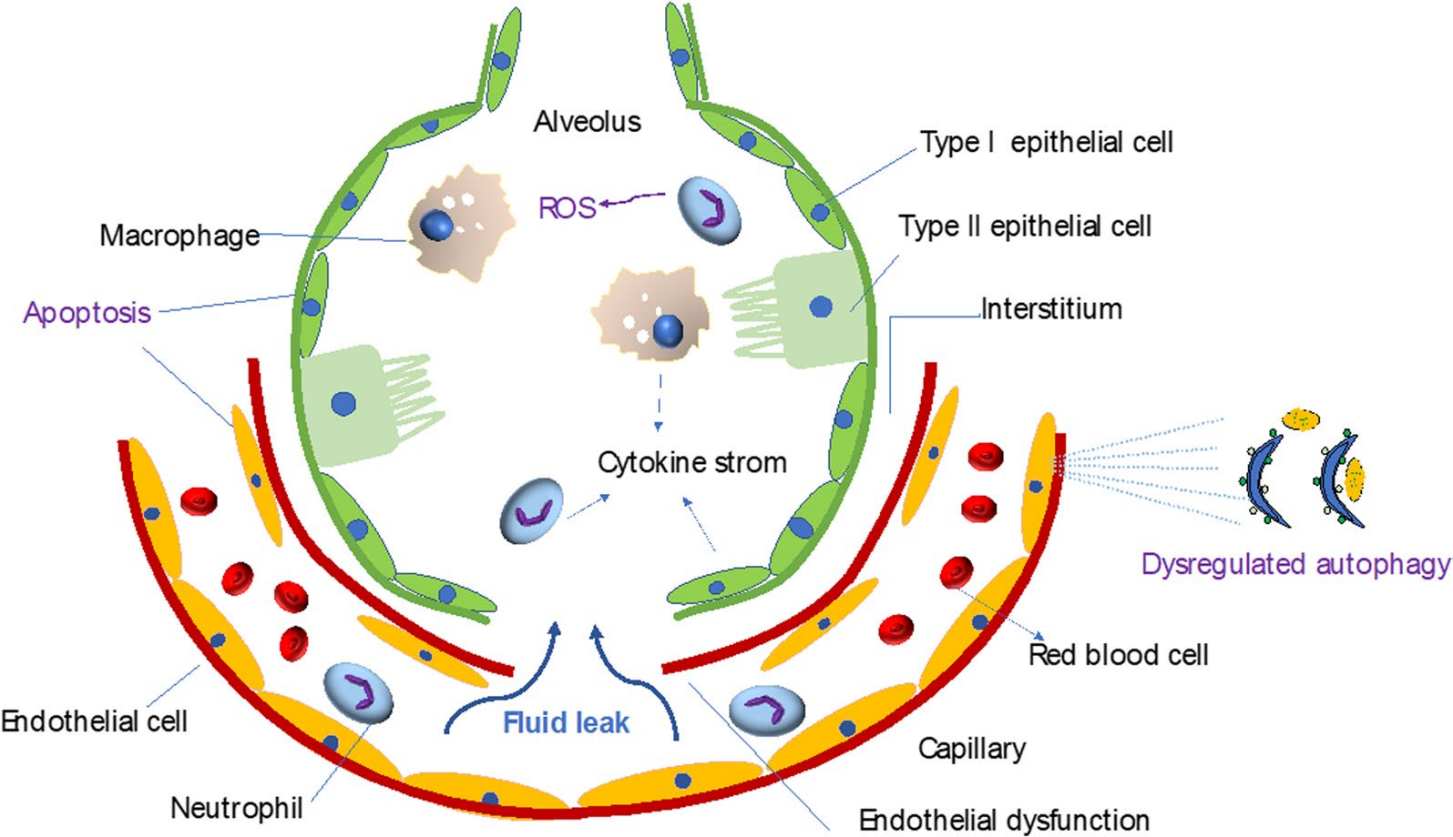
The pathogenesis of acute lung injury involves dysregulated inflammation and alveolar and endothelial barrier dysfunction

ALI/ARDS is characterized by a proteinaceous alveolar exudate, lung edema, endothelial and epithelial injury induced by dysregulated inflammation, and the destruction of alveolar/capillary barrier. It is also accompanied by an influx of neutrophils into the interstitium and bronchoalveolar space (Grommes and Soehnlein 2011) (Fig. 1). Based on the severity of hypoxemia, the 2012 Berlin Conference proposed the following three categories of ARDS: mild (200 mm Hg < PaO_2_/FiO_2_ ≤ 300 mm Hg), moderate (100 mm Hg < PaO_2_/FiO_2_ ≤ 200 mm Hg), and severe (PaO_2_/FiO_2_ ≤ 100 mm Hg) (Ranieri et al. 2012). ALI can be caused by a plethora of direct or indirect insults to the lung (Sharp et al. 2015). Direct factors include severe pulmonary infection, pulmonary embolism, and lung contusion, which can lead to serious alveolar lesions, whereas indirect factors include sepsis, trauma, massive transfusion, pancreatitis, fat embolism, and major surgery, which can initially trigger uncontrolled systemic inflammation, followed by multiple inflammatory cell infiltration and eventually, vascular endothelial injury (Bayat and Sachs 2012; Su et al. 2012) The pathogenesis of ALI includes a dysregulated inflammatory response, excessive oxidative stress, and dysregulated cell apoptosis and autophagy.

### Inflammation

An inflammatory response is the physiological response of the body to various stimuli and damages. However, uncontrolled inflammation in the lungs or the whole body contributes to the main pathogenesis of ALI/ARDS (Fan and Fan 2018). Different types of cells in the lung tissues, including neutrophils, macrophages, endothelial cells (ECs), and alveolar epithelial cells (AECs), participate in the inflammatory process; these are accompanied by the release of a cascade of inflammatory mediators from inflammatory cells (Herold et al. 2013; Nieman et al. 2015).

Neutrophils are the first to be recruited to the site of inflammation, and neutrophil recruitment into the lung is characteristic of ALI. Generally, neutrophils have a potent antimicrobial armor that includes oxidants, proteinases, and cationic peptides. However, the dysregulated release of these microbicidal compounds into the extracellular space can also paradoxically damage host tissues (Grommes and Soehnlein 2011).

Macrophages are highly plastic cells. The common inflammatory response involves the accumulation of proinflammatory M1 macrophages at the site of injury, followed by the appearance of anti-inflammatory/wound repair M2 macrophages; the balance in the activity of these macrophage subpopulations determines the outcome of the pathogenic response (Laskin et al. 2019). Macrophages recognize pathogen-associated molecular patterns (PAMPs) and trigger innate immune responses to activate host defenses (Wu et al. 2015), thereby playing an important role in the pathogenesis of ALI/ARDS by modulating inflammatory responses and repairing damaged lung tissues (Huang et al. 2018).

The pulmonary endothelium is an active continuous monolayer comprising ECs that internally line the blood vessels and mediates key processes in lung homeostasis. ECs play a major role in ALI/ARDS pathogenesis by altering hemostasis, weakening barrier function, and mediating intercellular signalling (Orfanos et al. 2004). EC dysfunction is also an important feature of hospitalized patients with COVID-19. In patients with COVID-19, the level of von Willebrand factor, which is a circulating adhesive glycoprotein secreted by ECs, is considerably elevated (Panigada et al. 2020), directing both endothelial infection with SARS-CoV-2 and the indirect damage caused by inflammation in COVID-19-associated coagulopathy (Iba et al. 2020). In addition, SARS-CoV-2 binds to and downregulates angiotensin-converting enzyme 2, resulting in remarkably increased vascular permeability in the lungs (Amraei and Rahimi 2020). Moreover, a common complication of COVID-19 is the “cytokine storm.” Cytokines can cause vascular leaks in the lung alveolar–endothelial interface and promote ALI (Vassiliou et al. 2020).

AECs can be categorized into two types: types I and II. Type I AECs are involved in facilitating gaseous exchange and can recognize pathogens. Type II AECs serve as innate immune cells and secrete surfactant proteins on their apical side. AECs are also potent regulators of the primary immune response against invading pathogens and act by releasing various immune mediators, such as antimicrobial peptides and cytokines, and by directly interacting with macrophages and neutrophils. However, the prolonged activation of AECs may harm the host through the release of a large amount of proinflammatory cytokines and chemokines and increase apoptosis (Hippenstiel et al. 2006; Kumar 2020).

There are several signal transduction pathways modulating the inflammatory process in ALI/ARDS such as nuclear factor kappa-B (NF-κB); the nucleotide-binding oligomerization domain-, leucine-rich repeat, and pyrin domain-containing 3 (NLRP3); mitogen-activated protein kinase (MAPK); and Toll-like receptor (TLR) signaling. NF-κB is considered the master regulator of inflammatory responses. It activates a series of proinflammatory transcriptional programs to shape the inflammatory response. Animal studies have established that this transcription factor is an important mediator in ALI/ARDS (Rahman and Fazal 2011). In addition, the high-mobility group box-1 protein (HMGB1), a late inflammatory mediator, can induce ALI by promoting NF-kB nuclear translocation, which can result in the release of inflammatory cytokines that further promote the release of HMGB1, resulting in a positive feedback loop that amplifies the inflammatory cascade (Entezari et al. 2014; Lee et al. 2018). The NF-κB pathway offers a variety of potential molecular targets for therapeutic intervention, and agents aimed at modulating the NF-κB pathway may alleviate ALI/ARDS (Wright and Christman 2003).

The NLRP3 inflammasome can be activated by the assembly of the NLRP3/apoptosis-associated speck–like protein/pro-caspase 1 protein complex, leading to the release of interleukin (IL)-1β (Martinon et al. 2002; Gross et al. 2011). Both the NLRP3 inflammasome and IL-1β mediate inflammation and contribute to inflammasome-associated pyroptosis (a mode of cell death) during ALI and ARDS (Ganter et al. 2008; Lee et al. 2016). NLRP3 activation is also involved in the pathogenesis of severe COVID-19, particularly in the formation of a cytokine storm. Therefore, targeting this pathway may provide insights for the treatment of severe COVID-19 (Freeman and Swartz 2020; Saeedi-Boroujeni et al. 2021).

p38 MAPK involves cell growth, differentiation, proliferation, migration, apoptosis, and inflammation (Pearson et al. 2001) and is upregulated in ALI (Ma et al. 2015; Xiong et al. 2016). Blocking of p38 MAPK signaling pathway alleviated the expression of proinflammatory cytokines and NLRP3 inflammasome in LPS-induced ALI (Li et al. 2018), bearing a potential for ALI therapy.

TLRs are an ancient evolutionarily family of pattern recognition receptors that play a central role in immune response by identifying PAMPs from pathogens and damage-associated molecular patterns from dying or injured cells. In lipopolysaccharide (LPS)-induced ALI, LPS binds to Toll-like receptor 4 (TLR4) and stimulates the TLR4-dependent inflammatory responses, including the activation of the TLR4/MyD88/NF-kB and TLR4/TRIF/IRF3 pathways (Togbe et al. 2007; Kuzmich et al. 2017). Blocking the TLR4 pathway is a potential strategy to alleviate LPS-induced ALI.

### Oxidative stress

Reactive oxygen species (ROS) include free radicals, such as **·**OH, superoxide anion radicals (O_2_·^−^), and non-free radical species such as singlet oxygen (^1^O_2_) and hydrogen peroxide (H_2_O_2_). They are generated inside the body by aerobic organism as a byproduct of energy metabolism through oxidative phosphorylation (Sies 2015). Normally, there are antioxidant defense systems in the cells that protect the biological systems from free radical toxicity such as superoxide dismutase (SOD), catalase (CAT), glutathione peroxidase (GSH-Px), and heme oxygenase-1 (HO-1) (Birben et al. 2012; Kellner et al. 2017). The dysregulation of ROS due to an imbalance in ROS generation and local antioxidant defenses results in oxidative stress, which causes oxidative damage to proteins, lipids, and nucleic acids (Ornatowski et al. 2020). During the pathogenesis of ALI/ARDS, the activation of neutrophils sequestered in pulmonary circulation can result in the release of free radicals and ROS, which leads to endothelial dysfunction and disruption, and is responsible for the principal clinical manifestations of this syndrome (Chabot et al. 1998). Recent studies suggest that oxidative stress plays an important role in viral infections, such as those caused by severe acute respiratory syndrome coronavirus and SARS-CoV-2, particularly in the more critical ARDS phase of infection (Cecchini and Cecchini 2020; Muhoberac 2020; Ntyonga-Pono 2020).

The transcription factor nuclear factor erythroid 2-related factor 2 (Nrf2) is a major regulator of the cytoprotective antioxidative protein expression products. The activation of Nrf2 signaling plays an essential role in preventing injury induced by oxidative stress to cells and tissues. The redox balance maintained by Nrf2 is important for the airways, and Nrf2 activation confers protective effects on various lung disorders including ALI/ARDS (Liu et al. 2019; Lee et al. 2021). Moreover, Nrf2 contributes to the regulation of the HO-1 axis and NF-κB pathway, both of which are potent anti-inflammatory targets. Nrf2 also participates in macrophage metabolism and the expression of inflammatory mediators (Saha et al. 2020).

Therapeutic intervention using reductants and chelators could be employed for ALI/ARDS. In both in vitro and in vivo experimental models, natural anti-oxidants, such as curcumin and garlic extract, decreased inflammation by activating Nrf2 and inducing Nrf2-regulated gene expression. However, these therapies remain to be translated into actual changes for patients with ARDS, and systematic studies must be conducted in human trials (Patel et al. 2018). Recently, vitamin C has been studied as a potential antioxidant to treat sepsis. Patients with sepsis have been found to be deficient in vitamin C, and high-dose intravenous vitamin C administration had a dose-dependent effect on the prevention of multiorgan failure and ARDS. However, considering its safety profile, current treatment is only justified for compassionate use (Kashiouris et al. 2020).

### Dysregulated autophagy and apoptosis

Autophagy is a highly conserved proteostatic process involving the degradation of cellular components such as lipids and misfolded proteins. This process helps maintain cellular and tissue homeostasis in response to numerous cellular stressors (Wang et al. 2019a). Autophagy plays a protective role in ALI/ARDS triggered by LPS, sepsis, and hyperoxia by regulating inflammatory-oxidative stress, apoptosis, pathogen clearance mechanisms, and balancing immune regulation, thereby avoiding recurrent exacerbations and disease progression in lungs (Yuan et al. 2012; Deretic et al. 2013; Junkins et al. 2013; Liu et al. 2013a).

Dysfunctional autophagy gives rise to various pathological states (Zhu et al. 2017; Mo et al. 2018). Recently, evidence suggested that SARS-CoV-2 inhibits autophagolysosomal formation and autophagy flux with impaired viral clearance and immune dysfunction. Therefore, targeting autophagy may prevent the replication of SARS-CoV-2 as a treatment for COVID-19 and allow a better tuning of inflammatory responses (Pehote and Vij 2020).

Autophagy is a complex process that can be harmful in excess. For instance, LPS-induced autophagy of alveolar macrophages in ALI promotes their transformation from type M2 to M1, which enhances apoptosis (Qiu et al. 2021). In addition, inhibition of autophagy in type II AECs is associated with alleviated inflammation and apoptosis (Zhang et al. 2015). Studies have found that autophagy in the septic lung represents a protective response; however, excessive accumulation of autophagosomes may play a maladaptive role in the late stage of sepsis, thus leading to ALI. Therefore, autophagy flux is a novel therapeutic target for the management of sepsis-induced ALI (Lo et al. 2013).

### Apoptosis and pyroptosis in ALI

Apoptosis is important in developmental biology and tissue remodeling during repair (Majno and Joris 1995). It is triggered by the two following fundamental signaling pathways: the extrinsic death receptor-mediated pathway and the intrinsic mitochondria-dependent pathway (Olson and Kornbluth 2001; Thorburn 2004).

Emerging evidence has suggested that the upregulation of pulmonary cell apoptosis is a pathophysiological consequence of various environmental stresses such as hypoxia, hyperoxia, oxidants, and LPS; these contribute to the initiation and progression of ALI and ARDS (Chopra et al. 2009). For instance, a clinical trial showed that excessive pulmonary EC apoptosis damages endothelial integrity and subsequently results in pulmonary endothelial barrier dysfunction, which has been observed in patients with severe ARDS (Abadie et al. 2005). Animal studies have demonstrated that inflammation and apoptosis are interrelated. For instance, p38 MAPK activation is linked to the initiation of the apoptotic cascade and plays a critical role in the development of apoptosis and pulmonary vascular permeability (Gill et al. 2015). In addition, increased apoptosis of AECs and alveolar macrophages can be observed, which results in the release of tumor necrosis factor-α (TNF-α) and transforming growth factor-β1, resulting in inflammation and progression from ARDS to fibrosis (Chapman 1999; Yang et al. 2018; Cui et al. 2020; Zhou et al. 2020a). Therefore, interventions targeting apoptosis may be instrumental for ALI/ARDS therapy.

Pyroptosis is a form of programmed necrosis triggered by inflammasomes, which detect cytosolic perturbations and drive the activation of caspase-1 or caspase-11/4/5. This causes cleavage of gasdermin D, separating its N-terminal pore-forming domain from the C-terminal repressor domain. Thereafter, the pore-forming domain oligomerizes, forming large pores in the membrane and driving swelling and membrane rupture (Kovacs and Miao 2017). Pyroptosis can occur in different cell types in ALI in a mouse model, including macrophages, neutrophils, and ECs, and plays an important role in the development of ALI (Liu et al. 2021). Inflammasomes are indispensable in the process of caspase-induced pyroptosis. The NLRP3 inflammasome is the most studied inflammasome, and it is involved in the activation of caspase-1 in the caspase-1-dependent pyroptosis pathway. At present, the inhibition of the NLRP3 inflammasome is an attractive and effective treatment for ALI caused by various pathogenetic factors such as LPS-induced ALI, ventilator-induced lung injury (VILI), and hyperoxia-induced ALI (HILI) (Kuipers et al. 2012; Hong et al. 2019; Wang et al. 2019b).

## Biological effects of molecular hydrogen

The potential mechanisms underlying the biological effects of molecular hydrogen remain unclear. Molecular oxygen may exert anti-inflammatory and antioxidant activities and modulate autophagy pathways and cell death pathways (apoptosis and pyroptosis) (Table 1).

**Table 1:** The proposed biological effects of molecular hydrogen

Biological effects	Main mechanisms	References
Anti-inflammation	Inhibits IL-1β, IL-6, TNF-α, HMGB-1, ICAM-1; Inhibits M1 polarization, increases M2 polarization of macrophages; Inhibits the recruitment of neutrophils to lesion sites, inhibits adhesion of polymorphonuclear neutrophils to ECs; The involved pathways: NF-κB, NLRP3, TLR4, Nrf2	Buchholz et al. (2008), Kawamura et al. (2013), Xiao et al. (2013), Ren et al. (2014), Chen et al. (2015b), Liu et al. (2015a), Shi et al. (2015), Xie et al. (2015), Zhai et al. (2015), Tian et al. (2017), Ning et al. (2018), Yu et al. (2019), Zou et al. (2019), Ming et al. (2020), Yang et al. (2020), Qiu et al. (2021)
Anti-oxidation	Directly scavenges **·**OH and ONOO^−^; activates Nrf-2/ HO-1; upregulates the expression of SOD, CAT, GSH-Px, downregulates NADPH oxidase; hormesis through increasing ROS	Ohsawa et al. (2007), Yu and Zheng (2012), Shinbo et al. (2013), Hirayama et al. (2018), Zhao et al. (2019)
Modulates autophagy	Promotes autophagy when autophagy is insufficient; inhibits autophagy when excessive autophagy disrupts cell homeostasis	Zhang et al. (2015), Yao et al. (2019), Chen et al. (2020a), Zhuang et al. (2020), Qiu et al. (2021)
Modulates cell death	Antiapoptosis: inhibiting Bax, caspase-3, and caspase-8, upregulating Bcl-xl and Bcl-2 Inhibits pyroptosis by inhibiting oxidative stress, NLRP3 and mitoK_ATP_/ERK1/2/p38 MAPK signaling pathways	Cai et al. (2008), Kawamura et al. (2010), Du et al. (2016), Nie et al. (2021), Zhang et al. (2021)

### Anti-inflammatory action

Molecular hydrogen exhibits anti-inflammatory activities in various injury models. In animal experiments, it downregulates the expression of proinflammatory and inflammatory cytokines, such as IL-1β, IL-6, TNF-α, HMGB-1, and intercellular cell adhesion molecule-1 (ICAM-1) (Buchholz et al. 2008; Tian et al. 2017). It also modulates the function of various inflammatory cells during inflammation, e.g., in LPS-induced injury, molecular hydrogen treatment substantially inhibited the increase in M1 cells, increasing M2 polarization of macrophages (Ning et al. 2018; Qiu et al. 2021); molecular hydrogen can inhibit the recruitment of neutrophils to lesion sites (Liu et al. 2015a), particularly inhibiting the adhesion of polymorphonuclear neutrophils to ECs to modulate the permeability of the vascular endothelium (Xie et al. 2015).

The anti-inflammatory effect of molecular hydrogen may involve inhibiting several inflammatory pathways, including the NF-κB pathway. Molecular hydrogen inhibits NF-κB activity, decreasing inflammatory factors in a variety of pathological models in rats or mice, such as cecal ligation and puncture-induced ALI, hepatectomy-induced postoperative cognitive dysfunction, ovalbumin-induced asthma, smoke inhalation injury, sodium taurocholate-induced acute pancreatitis induced acute renal injury (Xiao et al. 2013; Chen et al. 2015b; Shi et al. 2015; Zhai et al. 2015; Tian et al. 2017). HRS downregulated the expression of NLRP3 that is involved in lung and limb and intestinal I/R injuries, as well as in acute pancreatitis in rats (Ren et al. 2014; Zou et al. 2019; Yang et al. 2020). Molecular hydrogen inhibits the TLR4-mediated inflammatory pathway, improving hyperglycemia in rats with type 2 diabetes mellitus (Ming et al. 2020). It also regulates Nrf2 pathways, inhibiting oxidative stress-induced inflammatory lung disease in rats (Kawamura et al. 2013).

### Antioxidative action

Molecular hydrogen can directly scavenge the strong oxidants **·**OH and ONOO^−^ (Ohsawa et al. 2007; Shinbo et al. 2013). Owing to its intrinsic characteristics, such as nonpolarity and a small molecular weight, molecular hydrogen can easily penetrate the membrane and accumulate in the lipid phase; however, it does not affect normal metabolic redox reactions. In addition, molecular hydrogen can indirectly reduce oxidative stress by regulating gene expression. For instance, it can activate Nrf-2, enhancing the expression of HO-1, which is a downstream antioxidant of Nrf-2. It can also upregulate the expression of SOD, CAT, and GSH-Px and downregulate the expression of nicotinamide adenine dinucleotide phosphate (NADPH) oxidase in rats (Yu and Zheng 2012; Zhao et al. 2019).

In most cases, molecular hydrogen demonstrated an antioxidative activity. However, a clinical trial showed that inhaling a mixture of 1.2–1.4% molecular hydrogen–air increased urinary 8-hydroxy-2′-deoxyguanosine, a marker of oxidative stress, levels. This phenomenon of increasing ROS is associated with hormesis (Hirayama et al. 2018). In the process of hormesis, mitochondrial stress activates signaling pathways in cells to make them less susceptible to oxidative damage (Yun and Finkel 2014). The effect of increasing ROS on molecular hydrogen is similar to that induced by short and mildly strenuous exercise, and this may facilitate activating the Nrf2 anti-oxidative pathway (Hirayama et al. 2018).

### Action on autophagy

Besides having a direct anti-inflammatory effect, molecular hydrogen can modulate autophagy by downregulating the expression of NF-κB and MAPK and upregulating HO-1 activity (Liu and Zhang 2017; Ohta 2021; Slezak et al. 2021), suggesting that there is an intimate crosstalk between autophagy, inflammation, and ROS.

Molecular hydrogen promotes autophagy in conditions in which it is activated but not sufficient to overcome stress. For instance, in sepsis, an increase in endoplasmic reticulum stress (ERS) impaired autophagy. By activating the autophagy pathway, molecular hydrogen alleviated ERS and mitigated inflammation and organ injury in mice (Chen et al. 2020a). Similarly, through the mTOR-autophagy signaling pathway, molecular hydrogen activated autophagy and attenuated sepsis-induced neuroinflammation in both in vivo and in vitro experiments (Zhuang et al. 2020). Furthermore, molecular hydrogen can activate selective autophagy pathways such as mitophagy (sequestration of damaged mitochondria to lysosomes). Through phosphatase and tensin homolog-induced kinase 1 (PINK1)/Parkin-induced mitophagy, HRS could alleviate the inflammation response and apoptosis in in vivo and in vitro experiments in myocardial I/R injury (Yao et al. 2019). In these studies, autophagy represents an inducible response to stress and acts as an initial protective response to cell death (Ryter and Choi 2010). Impaired functioning of autophagy, such as impaired autophagosome-lysosome fusion, may cause the accumulation of autophagosomes (Lo et al. 2013). In such a situation, molecular hydrogen may help promote autophagy and increase the flow of autophagy to alleviate the harmful effect of stress.

In contrast, molecular hydrogen exerts an anti-autophagy effect when excessive autophagy disrupts cell homeostasis. For instance, in an LPS-induced ALI rat model, molecular hydrogen was shown to protect type II AECs by reducing the expression of autophagy-related proteins, thus decreasing the number of autophagosomes (Zhang et al. 2015). It also suppressed the autophagy of alveolar macrophages, thus regulating their polarization and apoptosis (Qiu et al. 2021). Molecular hydrogen may alter cell fate by regulating the balance between apoptosis and autophagy.

This dual effect of molecular hydrogen on autophagy may be explained by different levels of autophagy due to different stresses and treatment durations (Qiu et al. 2021). Autophagy is a double-edged sword; while it protects cells from stress-induced injury, when excessive, it disrupts cellular homeostasis and results in abnormal pathological conditions.

Detailed mechanisms of molecular hydrogen on autophagy still need to be clarified. Nonetheless, molecular hydrogen exerts protective effects by modulating autophagy and can harness autophagy pathways to maintain body hemostasis and protect cells from harmful stresses.

### Action on cell death

In most conditions, molecular hydrogen exerts antiapoptotic effects by modulating the expression of apoptosis-associated proteins, such as inhibiting the expression of the proapoptotic proteins, such as B-cell lymphoma-2-associated X-protein (Bax), caspase-3, and caspase-8, and upregulating the expression of antiapoptotic proteins, such as B-cell lymphoma-extra-large (Bcl-xl) and B-cell lymphoma-2 (Bcl-2) (Cai et al. 2008; Kawamura et al. 2010; Du et al. 2016). Additionally, molecular hydrogen can inhibit apoptosis by reducing inflammation and oxidative damage but activating autophagy (Yao et al. 2019; Qiu et al. 2020).

Molecular hydrogen can affect pyroptosis in various animal models by inhibiting oxidative stress and NLRP3-mediated pyroptosis, alleviating myocardial I/R injury in rats (Nie et al. 2021). Post-conditioning with hydrogen gas ameliorated subarachnoid hemorrhage-induced neuronal pyroptosis through the mitoK_ATP_/ERK1/2/p38 MAPK signaling pathway in rats (Zhang et al. 2021). In the lung, the inflation of 3% molecular hydrogen during the cold ischemia phase alleviated lung I/R injury by inhibiting pyroptosis and improving graft function in rats (Zheng et al. 2021a). It is conceivable that modulating pyroptosis is an important mechanism by which molecular hydrogen plays a protective role in ALI.

## Effect of molecular hydrogen on ALI

### Molecular hydrogen protects and restores epithelial and endothelial barrier function in the lung

Through its anti-inflammatory and antioxidative effects and by modulating autophagy and cell death, molecular hydrogen markedly alleviates tissue damage in the lung; in particular, it helps in restoring the lung barrier function.

As mentioned above, type I AECs are highly specialized for the gas exchange between alveoli and capillary blood in the lung. They are also involved in ion and water transport, the regulation of cell proliferation, and peptide metabolism signaling pathways in the peripheral lungs (Herzog et al. 2008). Type I AECs have ion channels and pumps for transcellular sodium transport (Johnson et al. 2002). Among these, aquaporins (AQPs) facilitate water flux across cell membranes and provide a transcellular route for water transport across epithelia (Wittekindt and Dietl 2019). Therefore, AQPs, particularly AQP1 and AQP5, are of tremendous significance in lung pathophysiology, such as in that of ALI (Yadav et al. 2020).

Molecular hydrogen has been shown to protect epithelial function in ALI in animal experiments. It alleviated AEC apoptosis (Shi et al. 2012), which potentially helps maintain the integrity of the epithelial barrier. It also protects AQP function. In our studies, we demonstrated that in LPS-induced ALI, the expression of AQP1 and AQP5 in the rat lung was downregulated. HRS treatment inhibited the activation of p38 MAPK and c-Jun N-terminal kinase, decreasing the inhibitory effect of LPS on AQP1 and AQP5 (Liu et al. 2016; Tao et al. 2016). These effects of molecular hydrogen help to keep the alveolar space relatively free from fluid overload, improving alveolar gas exchange to alleviate ALI.

**Fig. 2:**
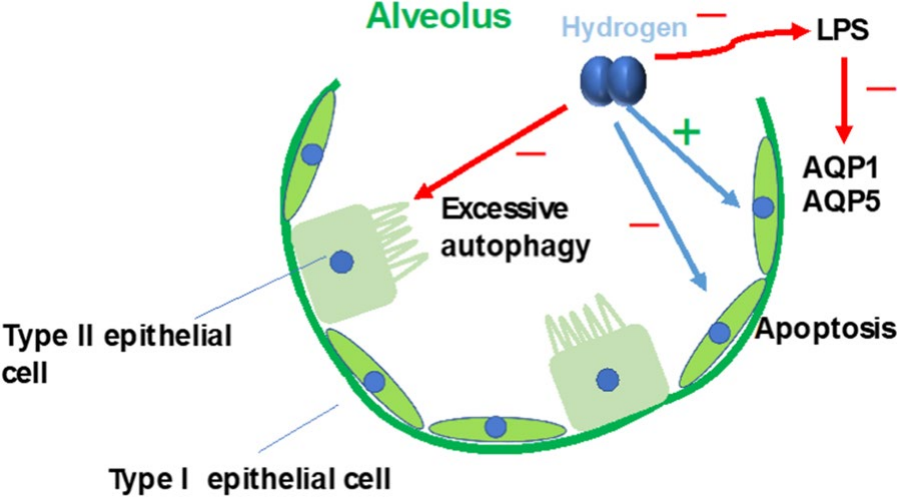
Protective effects of molecular hydrogen on AECs. Molecular hydrogen alleviates AEC apoptosis, decreasing the inhibitory effect of LPS on AQP1 and AQP5, and protects type II AECs in LPS-induced injury by inhibiting excessive autophagy

In addition, HRS can protect type II AECs in LPS-induced ALI by inhibiting excessive autophagy (Zhang et al. 2015), and type II AECs are involved in the metabolism of pulmonary surfactants and help maintain a sufficient respiratory surface area of the lungs at the end of expiration (Lopez-Rodriguez and Pérez-Gil 2014) (Fig. 2).

In addition to the epithelial barrier dysfunction, severe disturbances to the endothelial barrier are involved in ALI and ARDS (Ware and Matthay 2000). Restoring endothelial barrier function can effectively improve ALI outcomes. Endothelial permeability is maintained by transcellular and paracellular routes. The transcellular route is moderated by caveolae-mediated vesicular transport, whereas the paracellular route is supported by interendothelial junctions (including tight junctions (TJs) and adherens junctions (AJs)), which connect adjacent ECs with the monolayer (Komarova and Malik 2010). Occludin and vascular endothelial (VE)-cadherin are major components of TJ and AJ components, respectively. Occludin and claudins are linked to the zonula occludens and other protein complexes, mediating the interaction between adhesion molecules and actin filaments (Balda and Matter 2016). VE-cadherin maintains firm EC–EC junctions by binding its cytoplasmic domain to α-catenin and β-catenin (Dejana et al. 1995). In ALI, TJs and AJs in pulmonary microvascular endothelial cells (PMVECs) are damaged first, leading to a high endothelial permeability (Li et al. 2020). In addition to interendothelial junctions, another critical factor that influences endothelial permeability is the endothelial cytoskeleton, which maintains endothelial cell–cell adhesion by delicately regulating endothelial contractility and junctional organization. It is mainly regulated by small guanosine triphosphatases (GTPases) RhoA, Rac1, Cdc42) or Rap1 (Spindler et al. 2010). RhoA, the most studied protein in the Rho family, is activated in ALI, leading to the activation of Rho kinases (ROCK) and myosin light chain kinase, which phosphorylates the light chain of myosin and induces actomyosin contractility, leading to a weakening of endothelial cell–cell adhesion (Shen et al. 2010; Schnittler 2016; Yin et al. 2019).

In vitro studies showed that molecular hydrogen alleviates the hyperpermeability of the vascular endothelium in ALI, represented by decreasing the fluorescein isothiocyanate-dextran flux and increasing the transendothelial electrical resistance of EC (Xie et al. 2015; Yu et al. 2015). In LPS-induced endothelial injury, molecular hydrogen could increase the expression of VE-cadherin and influence its distribution (as manifested by comparatively even and complete at the cell joints (Yu et al. 2015), recover the reduced expression of occludin, and ameliorate the excessive expression of ROCK and RhoA (Yang et al. 2016; Li et al. 2020). Molecular hydrogen can also reduce the expression of ICAM-1 and its release from the cell, in addition to inhibiting the adhesion of monocytes to EC (Chen et al. 2015a; Xie et al. 2015; Yu et al. 2015).

**Fig. 3:**
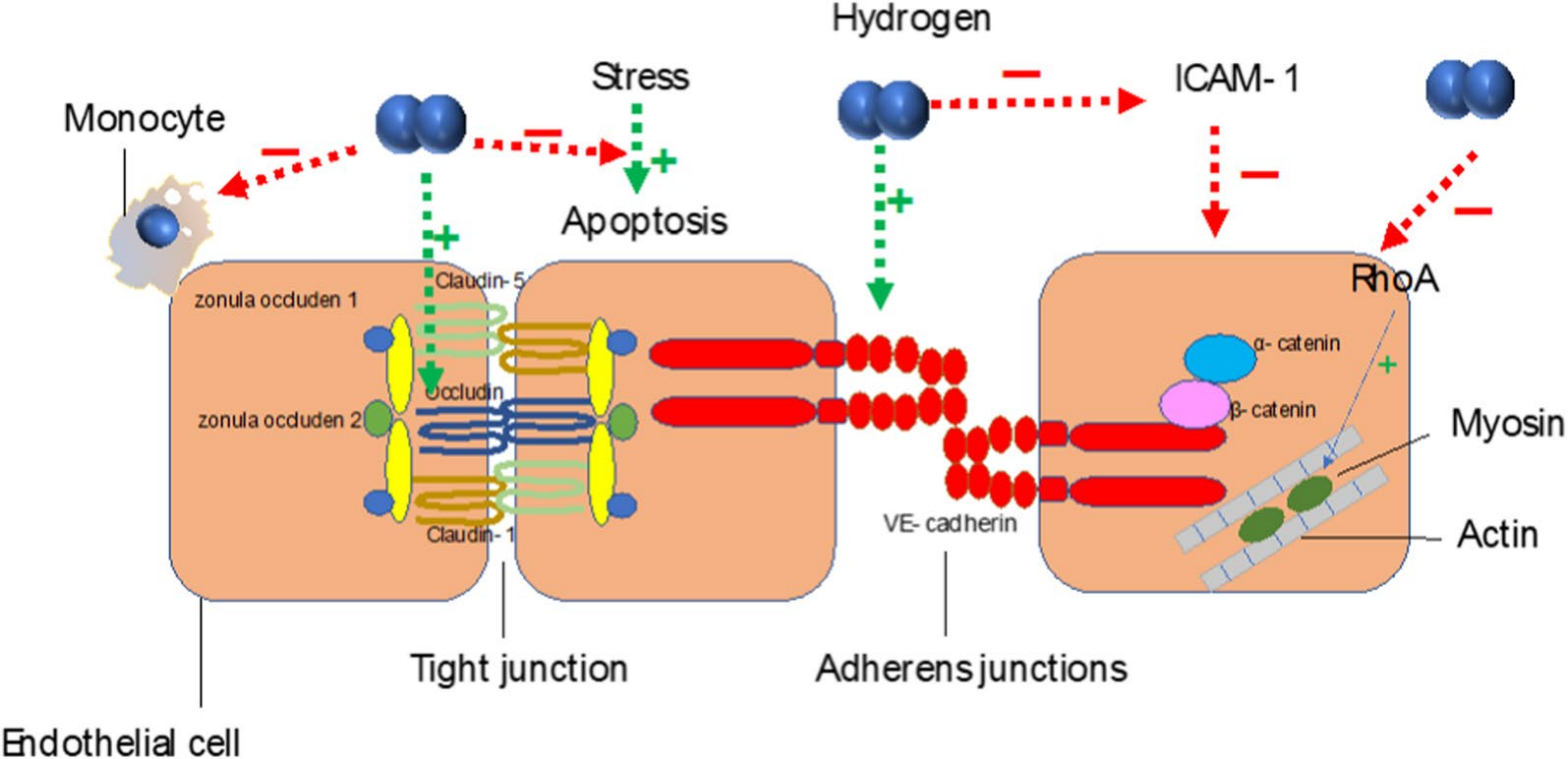
Molecular hydrogen alleviates the hyperpermeability of the vascular endothelium in ALI. The interendothelial junctions of ECs include tight junctions and adherens junctions. Occludin and claudins are linked to the zonula occludens. The cytoplasmic domain of VE-cadherin binds to α-catenin and β-catenin to firmly maintain EC–EC junctions. RhoA activates Rho kinases and myosin light chain kinase, which phosphorylates the myosin light chain, inducing actomyosin contractility and weakening endothelial cell–cell adhesion. Molecular hydrogen could increase the expression of VE-cadherin and occludin. It also inhibits the expression of RhoA, reduces the expression of ICAM-1 and its release from the cell, and inhibits the adhesion of monocytes to ECs

Physically, EC apoptosis is beneficial because it replaces damaged ECs infected with intracellular pathogens by healthy cells. However, under stress conditions, apoptosis may become a pathophysiological consequence of these stimuli (Chambers et al. 2018) and cause detrimental effects on the body. For instance, it disrupts the integrity of the endothelium and contributes to vascular leakage (Hotchkiss et al. 2002). Molecular hydrogen can prevent LPS-induced EC apoptosis by inhibiting the activity of RhoA or activating the Nrf2-mediated HO-1 pathway in in vitro experiments (Chen et al. 2015a; Li et al. 2020). In our study, we found that molecular hydrogen activated mTOR/transcription factor EB (TFEB)-mediated autophagy and inhibited HPMEC apoptosis in LPS-induced ALI (Fu et al. 2020). Through the inhibition of apoptosis, molecular hydrogen improves cell viability and suppresses the release of the cell adhesion molecules, such as vascular cell adhesion molecule-1 and ICAM-1, and pro-inflammatory cytokines, such as TNF-α, IL-1β, and HMGB1, potentially alleviating endothelial injury (Fig. 3).

### Molecular hydrogen confers a protective effect on extrapulmonary organs

ALI/ARDS is a severe lung disease involving the respiratory system and extrapulmonary distal organs (Del Sorbo and Slutsky 2011). For instance, although COVID-19 is well known for causing a substantial respiratory pathology, it can also cause thrombotic complications, neurologic illnesses, myocardial dysfunction and arrhythmia, acute coronary syndromes, acute kidney injury, hepatocellular injury, hyperglycemia and ketosis, and dermatological complications (Gupta et al. 2020). An intimate crosstalk occurs between the lungs and other organs that coordinate to provide the host immunity against infection and maintain homeostasis (Wang et al. 2021b). It was concluded that the predominant cause of death in patients with ARDS is not severe hypoxemia but multiple organ failure (Del Sorbo and Slutsky 2011); this highlights the importance of protecting extrapulmonary organs.

Molecular hydrogen has preventive and therapeutic effects on various organs. Its action in sepsis-induced lung injury and multiple organ dysfunction in animal experiments is most well studied. Molecular hydrogen reduces the damage to various organ functions and improves the survival rate (Xie et al. 2014; Zheng and Zhu 2016; Qiu et al. 2019; Qi et al. 2021), which may alleviate ALI. In sepsis-induced brain injury, molecular hydrogen can attenuate the disruption of the blood–brain barrier and decrease its permeability, thereby reducing sepsis-associated encephalopathy and improving cognitive function (Yu et al. 2020). Chronic exposure to molecular hydrogen prevents memory loss in patients with sepsis, whereas acute molecular hydrogen inhalation decreases neuroinflammation in memory-related areas and increases the activity of Nrf2 (Jesus et al. 2020). In sepsis-induced liver injury in mice model, molecular hydrogen activated the Fun14 domain-containing protein 1-induced mitophagy pathway, decreased the liver histological score, and decreased the levels of alanine aminotransferase and aspartate aminotransferase. Therefore, it may serve as an effective therapeutic strategy for sepsis-induced liver injury (Yan et al. 2019). In myocardial tissues with severe sepsis, molecular hydrogen gas treatment led to upregulated HO-1 and the expression of mitofusin-2 (Mfn2) and peroxisome proliferator-activated receptor-gamma coactivator-1*α*, while limiting severe sepsis-related mitochondrial dysfunction (Zhang et al. 2020) and restoring cardiac fatty acid oxidation by increasing cardiac energy in sepsis (Tao et al. 2015). In the kidney, molecular hydrogen remarkably reduces serum levels of blood urea nitrogen and Cr (Li et al. 2013), and it is considered useful in alleviating structural damage to the kidney and protecting renal function. In the septic intestine, the luminal administration of HRS prevented intestinal dysbiosis, hyperpermeability, and bacterial translocation, which is a major cause of multiple organ dysfunction syndrome in critical illness (Ikeda et al. 2018). Collectively, these results suggest that in addition to its direct protective effects on the lungs, molecular hydrogen can indirectly protect lung tissues via a mechanism that underlies its protective effects on other tissues and organs.

## Application of molecular hydrogen in ALI caused by various etiological factors

### Sepsis-induced ALI

Sepsis is a serious systemic inflammatory response syndrome that causes life-threatening organ dysfunction due to a dysfunctional host response to infection, which is a leading cause of ALI (Sessler et al. 1996). Molecular hydrogen has been widely used in studies focusing on sepsis in recent years and has yielded beneficial effects.

As mentioned above, oxidative stress plays an important role in the process of ALI/ARDS; however, data demonstrating the clinical improvements of antioxidant therapy remain limited. Molecular hydrogen has antioxidant capacity, and the main mechanism underlying this is the activation of the Nrf2/HO-1-dependent pathway, which reduces oxidative stress (Xie et al. 2012) and downregulates the expression and reduces the release of HMGB1 in the serum and lung tissues of septic mice.

Mitochondrial dysfunction plays an important role in sepsis-induced organ damage. In sepsis, molecular hydrogen therapy effectively improved mitochondrial function, reflected by the blocking of mitochondrial permeability transition pore openings, and an increase in mitochondrial-membrane potential and adenosine triphosphate (ATP) levels, respiration control ratio, mitochondrial–respiration complex activities, and Mfn2 expression along with a decrease in the histological score and dynamin-related protein 1 levels (Dong et al. 2018). Molecular hydrogen can also inactivate the NLRP3 inflammasome through the activation of autophagy and mitigation of mitochondrial dysfunction and cytokine release in septic mice (Chen et al. 2019).

When molecular hydrogen is combined with other therapies in sepsis, it can synergistically enhance their therapeutic effect. For instance, the combination of molecular hydrogen inhalation with early fluid resuscitation considerably reduced the increase in oxidative stress in the lung tissue, decreasing the degree of inflammation in septic rats (Liu et al. 2013b). The combination of inhaling molecular hydrogen and hyperoxia produced beneficial effects on organs in zymosan-induced septic mice (Hong et al. 2016). In addition, the combination of molecular hydrogen with propofol improved the survival rate of septic mice and reduced tissue damage (Hong et al. 2017).

LPS is a component of the outer membrane of gram-negative bacteria. It binds to TLR4, stimulating TLR4-dependent systemic inflammatory responses and triggering the host innate immune system. Molecular hydrogen treatment can reduce the degree of LPS-induced inflammation in lung tissues of experimental animals. It inhibits NF-κB signaling pathway-mediated inflammation and apoptosis in the lung (Xie et al. 2012), reduces p38 MAPK expression and inhibits p38 MAPK activation (Liang et al. 2012), inhibits the RhoA mediated pathway (Yang et al. 2016), and modulates autophagy through the mTOR/TFEB signaling pathway and the PINK1/Parkin mitophagy pathway (Fu et al. 2020; Chen et al. 2021). Recently, molecular hydrogen was found to activate thioredoxin 1 and decrease tissue factor expression, alleviating inflammatory and coagulation cascade reactions (Li et al. 2021). Through these mechanisms, molecular hydrogen considerably improved the survival rate of mice with LPS and reduced lung edema and hemorrhage, inflammatory cell infiltration, and inflammatory cytokine secretion.

### COVID-19

COVID-19 often manifests with mild cold-like symptoms, but severe disease with complications occurs in 15% of the cases (Zhou et al. 2020b). Respiratory failure occurring in severe cases is characterized by a systemic inflammatory reaction with inflammatory cytokine release (Zhu et al. 2020). At present, the only available therapeutic remedies are restricted to alleviating the side effects caused by the virus, such as inflammation and pulmonary fibrosis, which are recognized as the first causes of death.

A high level of IL-6 closely correlates with SARS-CoV-2 infection and the vital signs of COVID-19 patients (Chen et al. 2020b; Liu et al. 2020a). Microbes are known to bind to TLR, inducing IL-1, a highly inflammatory mediator of fever and fibrosis (Conti et al. 2020). Moreover, SARS-CoV-2 infection causing ARDS involves the NLRP3 inflammasome pathway and the release of its products, the proinflammatory cytokines IL-6 and IL-1β (Freeman and Swartz 2020). Treatments inhibiting the expression of IL-6 and IL-1 and targeting the NLRP3 inflammasome pathway would be a viable approach to reduce SARS-CoV-2-induced inflammatory cytokine signaling. Accumulating evidence has also suggested that COVID-19 induces oxidative stress by producing a substantial ROS load, especially in the more critical ARDS phase (Muhoberac 2020). Therefore, interventions using reductants may help treat COVID-19.

Clinical trials and animal experiments showed that molecular hydrogen can attenuate inflammation and oxidative stress in the airways and lungs in infective pulmonary diseases by inhibiting the proinflammatory cytokines MCP-1, IL-6, and IL-1β and the NLRP3 pathway, as well as modulating the Nrf2 signaling pathway (Chen et al. 2019; Niu et al. 2020; Wang et al. 2020). Molecular hydrogen may provide an effective and novel adjuvant treatment against COVID-19 and may be beneficial in preventing a COVID-19-associated cytokine storm and multiple organ failure (Wang et al. 2021a).

### Ischemia/reperfusion (I/R)-induced ALI

I/R-induced injury is associated with serious clinical outcomes. Its pathophysiology involves ROS, aseptic inflammation, and cell death pathways. I/R-induced ALI usually occurs after cardiac bypass surgery and lung transplantation (den Hengst et al. 2010), leading to primary graft dysfunction. So far, no effective therapy is available to prevent I/R injury and treatment strategies are mainly restricted to supportive care. At present, data on the application of molecular hydrogen molecules in clinical I/R-induced ALI are limited, but studies in animal models suggest that molecular hydrogen can reduce oxidative stress, inflammation, and apoptosis and alleviate I/R injury in lung transplantation.

During I/R, mounting ROS stimulate tissue inflammation and mitochondrial dysfunction and induce NLRP3 inflammasome activation, eventually hampering cellular homeostasis (Minutoli et al. 2016). In lung transplantation in rat models, when donors are ventilated using hydrogen gas, hydrogen-induced HO-1 (Kawamura et al. 2011) and inflation with hydrogen gas during the cold ischemia phase inhibited pyroptosis (Zheng et al. 2021a).

Molecular hydrogen inhibits the inflammatory response pathways, including the p38 MAPK and NF-κB pathways, thereby decreasing the levels of IL-8, IL-1β, and TNF-α in the recipient lung and decreasing myeloperoxidase (MPO) levels in a PMVECs lung transplantation model and a rat lung transplantation model (Liu et al. 2015b; Zhang et al. 2018; Saito et al. 2020).

Molecular hydrogen could also considerably inhibit apoptosis in rat lung transplantation and inhibit the expression of proapoptotic proteins caspase-3 and caspase-8 in lung grafts, but activate the antiapoptotic proteins Bcl-2 and Bcl-xL, thus stabilizing the mitochondrial outer membrane and terminating the release of cytochrome c into the cytosol via the intrinsic apoptotic pathway (Kawamura et al. 2010; Liu et al. 2015b). Additionally, pretreatment of rat donor lungs with molecular hydrogen can induce several lung surfactant-related, ATP synthase, and stress-response genes (Tanaka et al. 2012).

### Hyperoxia-induced acute lung injury

The administration of oxygen in high concentration is required to maintain sufficient blood oxygenation in some critically ill patients, but prolonged exposure to it can result in HILI, even leading to respiratory failure. HILI is mainly caused by excessive ROS generated during hyperoxic conditions (Altemeier and Sinclair 2007). So far, there are no effective prevention or treatment methods for HILI. An animal study found that HRS ameliorated HILI by reducing oxidative stress and inflammatory cascades, inhibiting apoptosis in lung tissues (Sun et al. 2011). The mechanism involved the Nrf2/HO-1-dependent pathway, prolonging survival against lethal hyperoxia in rats (Kawamura et al. 2013). Another study found that molecular hydrogen could reduce HILI-related ERS by increasing a master regulator of ERS-silent information regulator type-1 (SIRT1) expression in rats (Sun et al. 2017).

To further understand the molecular mechanisms underlying molecular hydrogen effects in HILI, quantitative proteomics in vitro revealed that molecular hydrogen can protect AECIIs from hyperoxic injury by modulating a range of protein expression levels and biological processes, such as VEGFA, PDGFB, IGFBP3, EDN1, NADPH oxidase levels, and the coagulation cascade (Lu et al. 2018). These findings provide a theoretical basis for the clinical application of molecular hydrogen in treating oxygen toxicity.

### VILI

Ventilator support is often required in the intensive care unit for critically ill patients with respiratory failure, but it can also induce lung injury and even exacerbate it (Tremblay and Slutsky 2006). Through its antioxidant, anti-inflammatory, and antiapoptotic effects, molecular hydrogen effectively reduced the inflammatory response in VILI at the local and systemic levels in mice (Huang et al. 2010). Interestingly, molecular hydrogen increased the early activation of NF-κB, which correlated with elevated levels of the antiapoptotic protein Bcl-2 and decreased levels of Bax, and contributed to the epithelial protective effects of molecular hydrogen against apoptosis and the activation of the inflammatory signaling pathway during VILI in a mouse model (Huang et al. 2011).

### Radiation-induced lung injury

Radiation is used in medical diagnosis and cancer treatment. However, damage can also occur during radiation therapy. ROS generated in therapy are one of factors contributing to the side effects of radiation therapy (Ward 1988; Shao et al. 2014). Extensive studies have revealed that molecular hydrogen exerts radioprotective effects as an antioxidant and intracellular response modulator (Hirano et al. 2021). In the human lung epithelial cell line A549 undergoing irradiation, molecular hydrogen reduced the amount of radiation-induced ROS and apoptosis while enhancing cell viability (Terasaki et al. 2011). In an in vivo mouse model, molecular hydrogen similarly attenuated oxidative stress and apoptosis that act as measures of acute damage. It also reduced late damage-pulmonary fibrosis as observed on alleviated chest computed tomography, Ashcroft score, and type III collagen deposition (Terasaki et al. 2011).

### Burn-induced ALI

Major burns can cause massive tissue destruction and activate a cytokine-mediated inflammatory response, which leads to dramatic pathophysiological effects at sites both local to and distant from the burn (Bittner et al. 2015).

In an extensive rat model for burns, severe burns with delayed resuscitation caused impaired oxygenation and lung edema. Intraperitoneal HRS ameliorated these effects, substantially attenuated pulmonary oxidative products, and reduced the levels of pulmonary inflammation mediators and myeloperoxidase (Fang et al. 2011). Similarly, Qin et al. found that at an early stage of severe burns in mice, molecular hydrogen considerably alleviated the infiltration of inflammatory cells and improved pathological lesions of the lung tissue, thus increasing the survival rates of mice (Qin et al. 2017).

SIRT1 expression in rat lung after burn injury presented an increasing trend after a short period of suppression. SIRT1 might provide protection from burn-induced remote ALI by attenuating PMVEC apoptosis via p38 MAPK signaling; thus, SIRT1 exhibits a potential therapeutic effect on burn-induced ALI (Bai et al. 2015). Molecular hydrogen can increase SIRT1 expression in hyperoxia ALI. Therefore, although further investigation is needed, molecular hydrogen may modulate SIRT1 in burn-induced ALI.

### ALI induced by other causes

In seawater instillation-induced ALI in rabbits, molecular hydrogen inhalation alleviated histopathological changes and cell apoptosis; decreased the malondialdehyde content and MPO activity in lung tissues; decreased the levels of TNF-α, IL-1β, and IL-6 in bronchoalveolar lavage fluid; and markedly improved lung endothelial permeability. These protective effects may be related to the activation of the Nrf2 pathway (Diao et al. 2016).

In addition, molecular hydrogen attenuates gefitinib-induced exacerbation of naphthalene-evoked ALI (Terasaki et al. 2019) and oleic acid-induced ALI (Ying et al. 2017) in animal experiments. A combination of molecular hydrogen and hyperoxia improved the survival rate and reduced the organ damage in a zymosan-induced generalized inflammation mouse model. This combination showed a more marked beneficial effect against lung, liver, and kidney damage (Hong et al. 2016). It can be concluded that molecular hydrogen has a wide therapeutic application spectrum in the treatment of ALI caused by various pathogenetic factors. The application of molecular hydrogen in ALI is summarized in Table 2.

**Table 2:** Application of molecular hydrogen in ALI caused by various etiological factors

Etiological factors	Effect of molecular hydrogen	References
Sepsis-induced ALI	Inhibits HMGB1 by activating Nrf2/HO-1; improves mitochondrial function; decreases NLRP3 inflammasome through activating autophagy Inhibits NF-κB; inhibits p38 MAPK; inhibits RhoA; modulates mTOR/TFEB autophagy and PINK1/Parkin mitophagy; activates thioredoxin 1 and decreases tissue factor expression	Xie et al. (2010, 2012), Liang et al. (2012), Li et al. (2015, 2021), Yang et al. (2016), Dong et al. (2018), Chen et al. (2019, 2021), Yu et al. (2019), Fu et al. (2020)
COVID-19	Inhibits MCP-1, IL-6, IL-1β; inhibits NLRP3 pathway, modulates Nrf2 signaling pathway	Chen et al. (2019), Niu et al. (2020), Wang et al. (2020)
I/R induced ALI	Induces HO-1, inhibits p38 MAPK and NF-κB, decreases IL-8, IL-1β, TNF-α; inhibits apoptosis and pyroptosis	Kawamura et al. (2010, 2011), Liu et al. (2015b), Zhang et al. (2018), Saito et al. (2020), Zheng et al. (2021a)
HILI	Activates Nrf2/HO-1; increases SIRT1; protects AECIIs by modulating related proteins expression and biological processes	Kawamura et al. (2013), Sun et al. (2017), Lu et al. (2018)
VILI	Reduces inflammatory responses at local and systemic level; increases the early activation of NF-κB	Huang et al. (2010, 2011)
Irradiation-induced lung injury	Reduces ROS and apoptosis in A549 cells; reduces lung fibrosis	Terasaki et al. (2011)
Burn induced ALI	Attenuates oxidative stress and inflammation; may modulate SIRT1	Fang et al. (2011), Bai et al. (2015), Qin et al. (2017)
ALI induced by other causes	The protective effects are mainly by inhibiting oxidative stress, inflammation, apoptosis	Diao et al. (2016), Hong et al. (2016), Ying et al. (2017), Terasaki et al. (2019)

## Perspective

Molecular hydrogen is easily applicable because it exhibits remarkable efficacy on nearly all pathogenic states that are involved in inflammation and oxidative stress. In this study, we reviewed the protective effects and underlying mechanism of molecular hydrogen in ALI caused by different pathogenetic factors. The anti-ALI effects of molecular hydrogen were mainly attributed to its anti-inflammatory, antioxidative, antiapoptotic, and autophagy-modulating effects. However, the potential molecular mechanisms still need further exploration, and some results remain controversial, warranting further research. Although none have been found till date, it is unlikely that there are no adverse effects of molecular hydrogen. Additional research is required to confirm whether molecular hydrogen has undetected potential adverse effects to provide better clinical guidance.

Most current research is based on animal experiments; additional clinical trials are needed. Until now, inhalation of molecular hydrogen at a concentration of 1.3–4% has been employed as a form of treatment in clinical trials; however, the optimal dose of molecular hydrogen varies depending on the type of expected biological effect. Recently, a prospective, open-label, rater-blinded clinical pilot study in patients experiencing ST-elevation myocardial infarction demonstrated that molecular hydrogen gas inhalation during percutaneous coronary intervention is feasible and safe and may also promote left ventricular reverse remodeling at 6 months after ST-elevated myocardial infarction (Katsumata et al. 2017). Another multicenter, randomized, controlled clinical trial showed that the administration of a molecular hydrogen and oxygen mixture was superior to oxygen to improve symptoms in patients with acute exacerbation of chronic obstructive pulmonary disease (Zheng et al. 2021b).

For COVID-19, which has led to an urgent demand for therapeutic agents to alleviate and prevent the pandemic, many therapeutics and interventions have been investigated to combat the viral infection-induced inflammation and oxidative stress that contribute to its etiology and pathogenesis; however, these have not yielded any significantly improved treatment outcomes, In China, Nanshan et al. applied H_2_/O_2_ inhalation for the treatment of COVID-19 in more than 2000 patients and yielded positive and effective outcomes (Alwazeer et al. 2021). H_2_/O_2_ inhalation therapy can be performed in general wards or at home using portable H_2_/O_2_ generating and inhalation devices. This will ease the pressure on hospitals, reduce hospitalization time, and prevent severe illness caused by COVID-19. Currently, several clinical trials of molecular hydrogen on COVID-19 are registered in the Clinical Trial Registry (Alwazeer et al. 2021).

Till date, the reported benefits of molecular hydrogen therapy in patients with ALI are still limited to symptomatic descriptions. To expand the utility of molecular hydrogen therapy in ALI, further understanding of molecular hydrogen pharmacokinetics and its biological mechanisms of action will facilitate the clinical applications of this important molecule. We believe that this will generate standard clinical practice in the near future, and molecular hydrogen may provide strategies for ALI.

## Data Availability

Not applicable.

## Abbreviations

8-OHdG: 8-Hydroxy-2′-deoxyguanosine
AECs: Alveolar epithelial cells
AJ: Adherens junctions
ARD: Acute respiratory distress syndrome
ALI: Acute lung injury
AQPs: Aquaporins
ATP: Adenosine triphosphate
Bax: B-cell lymphoma-2-associated X-protein
Bcl-2: B-cell lymphoma-2
Bcl-xl: B-cell lymphoma-extra-large
CAT: Catalase
COVID-19: Coronavirus disease 2019
ECs: Endothelial cells
ERS: Endoplasmic reticulum stress
GSH-Px: Glutathione peroxidase
GTPases: Guanosine triphosphatases
HO-1: Heme oxygenase-1
HILI: Hyperoxia-induced ALI
HMGB1: High-mobility group box-1 protein
HRS: Hydrogen-rich saline
I/R: Ischemia/reperfusion
ICAM-1: Intercellular cell adhesion molecule-1
ICU: Intensive care unit
IL: Interleukin
LPS: Lipopolysaccharide
MAPK: Mitogen-activated protein kinase
Mfn2: Mitofusin-2
MPO: Myeloperoxidase
NADPH: Nicotinamide adenine dinucleotide phosphate
NF-κB: Nuclear factor kappa-B
NLRP3: Nucleotide-binding oligomerization domain-, leucine-rich repeat- and pyrin domain-containing 3
Nrf2: Nuclear factor erythroid 2-related factor 2
PAMPs: Pathogen-associated molecular patterns
PINK1: Phosphatase and tensin homolog-induced kinase 1
PMVECs: Pulmonary microvascular endothelial cells
ROCK: Rho kinases
ROS: Reactive oxygen species
SARS-CoV-2: Severe acute respiratory syndrome coronavirus 2
SIRT1: Silent information regulator type-1
SOD: Superoxide dismutase
TLR: Toll-like receptor
TNF-α: Tumor necrosis factor-α
TJ: Tight junction
VE: Vascular endothelial
VILI: Ventilator-induced lung injury
